# Cigarette smoke enhances Th-2 driven airway inflammation and delays inhalational tolerance

**DOI:** 10.1186/1465-9921-9-42

**Published:** 2008-05-20

**Authors:** Chris L Van Hove, Katrien Moerloose, Tania Maes, Guy F Joos, Kurt G Tournoy

**Affiliations:** 1 Department of Respiratory Diseases, Ghent University, Ghent, Belgium

## Abstract

**Background:**

Active smoking increases asthma severity and is related to diminished treatment efficacy. Animal models in which inhalation of both allergen and mainstream cigarette smoke are combined can help us to understand the complex interaction between both agents. We have recently shown that, in allergic mice, the airway inflammation can be cleared by repeated allergen challenge, resulting in the establishment of a state of inhalational tolerance.

**Methods:**

In this study, we assessed *in vivo *the impact of cigarette smoke on the efficacy and time course of this form of tolerance induction. We exposed sensitized mice to concurrent mainstream cigarette smoke and allergen (Ovalbumin- OVA) and measured the airway inflammation at different time points.

**Results:**

We first confirmed that aerosolized OVA administered for a prolonged time period (4–8 weeks) resulted in the establishment of tolerance. Concurrent OVA and smoke exposure for 2 weeks showed that tobacco smoke enhanced the Th-2 driven airway inflammation in the acute phase. In addition, the induction of the tolerance by repeated inhalational OVA challenge was delayed significantly by the tobacco smoke, since 4 weeks of concurrent exposure resulted in a more persistent eosinophilic airway inflammation, paralleled by a more mature dendritic cell phenotype. However, smoke exposure could not prevent the establishment of tolerance after 8 weeks of antigen exposure as shown by both histopathology (disappearance of the Th-2 driven inflammation) and by *in vivo *functional experiments. In these tolerized mice, some of the inflammatory responses to the smoke were even attenuated.

**Conclusion:**

Cigarette smoke enhances acute allergic inflammation and delays, but does not abrogate the development of tolerance due to prolonged challenge with inhaled antigen in experimental asthma.

## Background

Immune-mediated tolerance encompasses a number of mechanisms by which the immune system avoids unnecessary inflammatory responses, not only in face of auto-allergens but also to harmless environmental antigens [1]. It has been suggested that the beneficial and often long-lasting effects of allergen specific immunotherapy in patients with allergy are due to a stimulation of these immune-suppressive mechanisms [2]. We and others have found that prolonged exposure to aerosolized allergen in sensitized mice is accompanied with a disappearance of the eosinophilic airway inflammation [3-6] under certain conditions of antigenic stimulation, resulting in a state of inhalational tolerance [3,6]. Although a more persistent inflammation in experimental models of asthma can be obtained [7-10], the observation that the inflammation disappears under particular conditions of antigenic stimulation remains of potential importance, as it indicates that the vertebrate immune system has an inherent capacity to avoid unnecessary immunity and to restore homeostasis after an episode of allergen-induced airway inflammation. Studying the mechanisms involved in this phenomenon can provide us valuable new insights in the mechanisms regulating immune responses in the respiratory tract.

As a state of inhalational tolerance can be re-established *in vivo *[3,6], the question arises of its susceptibility and robustness in face of the numerous environmental factors that are known to aggravate the allergic condition [11,12]. One of the most important of such factors is mainstream cigarette smoke. To date, little is known about the effects of smoking on the efficacy of immunotherapy treatments in patients. Nevertheless, from animal models, we know that mainstream cigarette smoke has the potential to break primary inhalational tolerance to allergens in naïve animals [13] and to increase the systemic sensitization to surrogate and environmental allergens [14]. Several other studies have documented similar properties of second hand smoke or environmental tobacco smoke [15,16]. More conflicting data were reported on the effects of mainstream cigarette smoke in an ongoing acute Th-2 driven inflammatory response. While concurrent exposure to allergen and cigarette smoke was shown to aggravate allergic airway inflammation [17], tobacco smoke exposure following an initial period of allergen challenge, attenuated airway hyperresponsiveness and airway inflammation [14,18].

We hypothesized that mainstream cigarette smoke exposure could interfere with the induction of inhalational tolerance, thereby inducing a more persistent eosinophil-rich airway inflammation. This basic hypothesis seemed plausible from a theoretical point of view, since active smoking is associated with asthma exacerbations and reduced efficacy of treatment in humans [19] and mainstream cigarette smoke was able to break primary inhalational tolerance in naïve mice [13]. Active smoking could thus serve as an environmental factor that helps to explain why in patients with allergic asthma, the failure of immune tolerance persists. Therefore, we here analysed the effects of cigarette smoke exposure on the efficacy of inhalational tolerance induction in allergic mice.

## Methods

### Animals

Male C57BL/6 mice, 6 to 8 weeks old, were purchased from Harlan (Zeist, the Netherlands). All experimental procedures were approved by the local ethical committee for animal experiments (Faculty of Medicine and Health Sciences, Ghent University).

### Experimental Protocols

#### Allergen Protocol 1

OVA aerosol exposure

##### 1.1. Allergen Protocol 1A

Allergic airway inflammation

**Figure 1:**
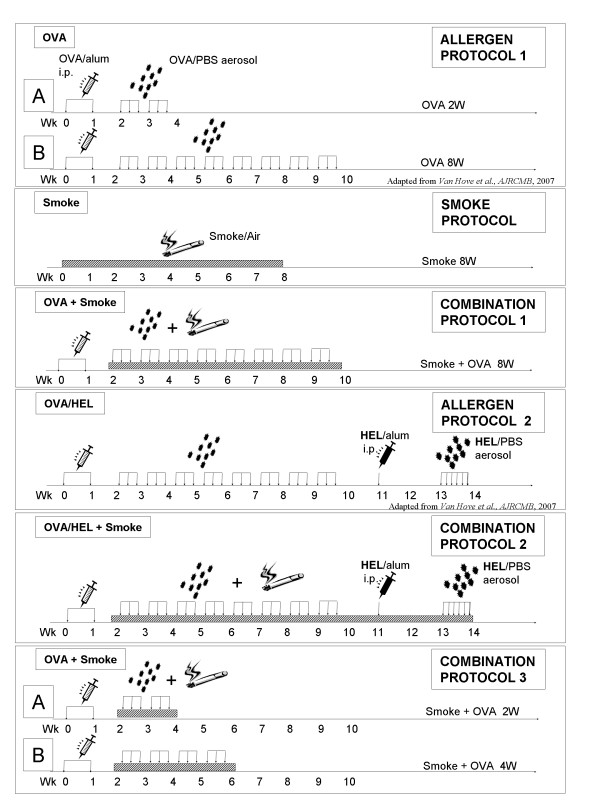
**Cigarette smoke and allergen exposure protocols**. **Allergen exposure protocols (*Allergen Protocols)****: Allergen Protocol 1A*: Exposure of sensitized C57BL/6 mice to PBS or OVA aerosols for 2 weeks. (n = 8 mice/group)*. Allergen Protocol 1B*: Prolonged PBS or OVA aerosol exposure in sensitized mice (8 weeks). (n = 8 mice/group). *Allergen Protocol 2: *OVA sensitized mice were exposed to OVA for 8 weeks ("chronic"), followed by HEL/alum immunisation and short-term ("acute") HEL aerosol challenge (OVA/HEL group). Control mice were exposed to PBS for 8 weeks, immunized with HEL/alum and challenged with either PBS (PBS/PBS group) or with HEL (PBS/HEL group). (n = 8–12 mice/group). **Smoke exposure protocol (*Smoke Protocol)****: *C57BL/6 mice are exposed to Smoke or Air for 8 weeks. (n = 6 mice/group). **Combined protocols (*Combination Protocols)*: ***Combination Protocol 1: *similar to *Allergen Protocol 1B*, combined with Smoke exposures or Air. (n = 8 mice/group). *Combination Protocol 2*: similar to *Allergen Protocol 2*, combined with or without Smoke. (n = 8 mice/group). *Combination Protocol 3A: *similar to *Allergen Protocol 1A*, combined with Smoke exposures or Air. (n = 8 mice/group). *Combination Protocol 3B: *4 weeks of OVA aerosol or PBS exposures in sensitized mice, combined with Smoke exposures or Air. (n = 8 mice/group). Vertical arrows = aerosol challenges (OVA, HEL or PBS). Stripped horizontal bars = period of Smoke or Air exposures.

Two groups of 8 mice were sensitized with 10 μg intraperitoneal (i. p.) OVA (Grade III; Sigma, St-Louis, MO) adsorbed to 1 mg Al(OH)_3 _on day 0 (d0) and d7. From d14 onward, the mice were exposed to aerosolized (Ultraschallvernebler Sirius Nova, Heyer Medizintechnologie, Bad Ems, Germany) OVA (1% wt/v) or PBS 30 min/d, 3 times a week for 2 weeks (Figure 1 – *Allergen Protocol 1A*).

##### 1.2. Allergen Protocol 1B

Tolerance

Two groups of mice (n = 8/group) were sensitized with i. p. OVA/alum on day 0 (d0) and d7. From d14 onward, the mice were subjected to prolonged OVA or PBS aerosol exposures for 8 weeks (Figure 1 – *Allergen Protocol 1B*).

#### Smoke Protocol

Mainstream cigarette smoke exposure *in vivo*

To show that exposure to mainstream cigarette smoke for 8 weeks is biologically active in C57BL/6 mice, two groups of 6 mice were exposed to either Smoke or Air for 8 weeks. Tobacco smoke exposures were performed in a plexiglass chamber (volume: 7500 cm^3^) with an inlet for pressured air (2.5 l/min). The chamber was connected to a smoking machine (D. Kobayashi, Washington University Medical Center, WA, USA). Exposures were performed four times a day, 5 days a week, with five Kentucky Reference cigarettes (2R4F, without filter) for six mice per exposure (Figure 1 – *Smoke Protocol*). The effects of this smoking protocol in mice at various other time points have been carefully characterized in earlier experiments by our laboratory[20]. Carboxyhemoglobin in the serum of mice exposed to this smoke protocol reached a non-toxic level of 8.3 ± 1.4% (compared with 1.0 ± 0.2% in air-exposed mice), which is similar to COHb blood concentrations of human smokers.

#### Combination Protocol 1

Smoke combined with long – term OVA aerosol exposure.

To evaluate the effect of cigarette smoke on the development of tolerance, four groups of mice (n = 8/group) were subjected to this tolerance induction protocol (OVA or PBS aerosols), combined with Smoke or Air. Smoke exposures were performed as described above in the *Smoke protocol*, with eight mice per exposure. Three times a week, smoke exposure was followed by OVA or PBS aerosols. The last smoke exposure took place 30 min before OVA or PBS aerosol. (Figure 1 – *Combination Protocol 1*).

#### Allergen Protocol 2

OVA and HEL aerosol exposures

To measure the immune responsiveness after chronic OVA exposure, OVA sensitized mice (n = 8–12/group), subjected to the tolerance protocol (8 wk OVA aerosols), were re-sensitized to a bystander allergen, Hen Egg Lysozyme (HEL, Sigma, St-Louis, MO; 10 μg HEL/alum i.p.) and re-challenged with daily HEL (1%) for 1 week ('OVA/HEL' group). OVA sensitized, PBS aerosol exposed mice were also re-immunized and challenged with HEL and were included as positive controls ('PBS/HEL' group). A negative control group ('PBS/PBS') was included as well (Figure 1 – *Allergen Protocol 2*).

#### Combination Protocol 2

Smoke combined with OVA and HEL aerosols

This experiment aimed to measure the immune responsiveness after chronic OVA and smoke exposure. OVA and Smoke or Air exposures were performed as in *Combination Protocol 1 *in two groups of mice (OVA/Smoke and OVA/Air groups; n = 8/group). The protocol was extended by subsequently re-sensitizing these mice to HEL (HEL/alum i. p.), and re-challenging them as described above. Meanwhile, the smoke exposures were continued in the test group ('OVA/HEL/Smoke' group), while remaining absent in the control group ('OVA/HEL/Air' group; Figure 1- *Combination Protocol 2*).

#### Combination Protocol 3

Smoke combined with short-term OVA aerosols

To evaluate effects of the smoke exposures on the time course of the tolerance induction, four groups of OVA sensitized mice (n = 8/group) were exposed to OVA or PBS aerosols for 2 and 4 weeks, combined with Smoke or Air. (Figure 1-*Combination Protocols 3A *and *3B *respectively).

### Broncho-alveolar lavage fluid (BALF): cellular analysis

Twenty-four hours after the last aerosol exposure, mice were sacrificed with i.p. pentobarbital (60 mg/kg; Sanofi, Libourne, France). BALF was taken by instillation of HBSS via a tracheal cannula. Three lavages with 0.3 ml HBSS followed by three lavages with 1 ml HBSS were performed. The recovered BALF of the first three fractions was centrifuged and the supernatant was used for cytokine detection. The cell pellet was then added to the rest of the lavage fluid, centrifuged, subjected to red blood cell lysis and resuspended for cell counts on cytospins (May-Grünwald/Giemsa).

### Tissue processing and preparation of lung single-cell suspension for flow cytometric analysis

Following BALF, the pulmonary and systemic circulation was rinsed with saline-EDTA to remove the pulmonary intravascular pool of cells. The right lung was thoroughly minced and incubated in digestion medium at 37°C containing DNase I (grade II from bovine pancreas; Boehringer Ingelheim, Germany) and collagenase type II (Worthington Biochemical Corp., Lakewood, NJ). After obtaining a single-cell suspension, samples were centrifuged and resuspended in PBS containing 10 mM EDTA. Finally, cells were subjected to red blood cell lysis, washed and kept on ice until labelling. Cell counting was performed with a Z2 Beckman-Coulter particle counter (Beckman-Coulter, Ghent, Belgium). Flow cytometric analysis of dendritic cells and T-lymphocytes was defined as detailed previously[20]. Briefly, mouse dendritic cells populations were identified as low autofluorescent, CD11c^high ^MHCII^+ ^cells. The maturation status of the dendritic cells was analysed by assessing the CD86 (B7.2) expression. Mouse T cell subpopulations were identified as CD3^+ ^and CD4^+ ^or CD8^+ ^cells. CD69 expression was analysed to assess the activation status of the T cells. Monoclonal antibodies used to identify mouse dendritic cells populations were biotinylated anti-CD11c and phycoerythrin (PE)-conjugated anti-IAb and anti-CD86. Rat IgG_2a_-PE was used as isotype control. Markers used for mouse T-cell subpopulations staining were CD3-APC, CD4-FITC, CD8-FITC and CD69-PE (activation marker). All other monoclonal antibodies were obtained from Becton Dickinson Pharmingen (BD, Erembodegem, Belgium), except anti-CD11c (N418 hybridoma; gift from M. Moser, Brussels Free University, Belgium). Flow cytometry data acquisition was performed on a duallaser FACSCalibur™ flow cytometer running CELLQuest™ software (BD, Mountain View, CA, USA). FlowJo software (Treestar Inc., Ashland, OR) was used for data analysis.

### Histology

After fixation of the left lung with 4% paraformaldehyde, slices from the left lobes were embedded in paraffin for histological analysis. Sections of 2 μm were stained with Congo Red, counter-stained with hematoxylin, to highlight eosinophils.

### Semi-quantitative analysis of peribronchial inflammation

The slides were coded and the peribronchial (and perivascular) inflammation was graded in a blinded fashion using a reproducible scoring system [21]. A value from 0 to 3 was adjudged to each tissue section scored. A value of 0 was given when no inflammation was detectable, a value of 1 for occasional cuffing with inflammatory cells, a value of 2 when most bronchi were surrounded by a thin layer (1 to 5 cells) of inflammatory cells and a value of 3 when most bronchi were surrounded by a thick layer (>5 cells) of inflammatory cells. As 5–7 tissue sections per mouse were scored, inflammation scores could be expressed as a mean value per animal and could be compared between groups.

### Lymph node cultures

Paratracheal and parathymic intrathoracic lymph nodes were removed. These mediastinal lung-draining lymph nodes were harvested into Tissue Culture Medium (TCM) containing tubes and digested enzymatically (collagenase/DNase) to obtain a single-cell suspension. Cells were cultured in TCM in a flat-bottom, 96 well plate (BD) alone or with 3 μg OVA or HEL, at a density of 8 × 10^5 ^cells per well. After 5 days of culture, supernatants were harvested and used for cytokine measurements.

### IgE, cytokines and chemokines (ELISA)

Total, OVA- and HEL-specific serum IgE was measured with ELISA using coated microtiter plates and biotinylated polyclonal rabbit anti-mouse IgE (S. Florquin, ULB, Brussels, Belgium). OVA- and HEL- specific IgE are expressed in arbitrary units (U) per ml serum. ['OVA-IgE Units' (OVA-U) or 'HEL-IgE Units' (HEL-U)/ml]. One 'OVA-IgE Unit' was defined as a 1/100 dilution of an internal standard serum pool obtained from OVA (10 μg)/alum (1 mg) sensitized mice. One 'HEL-IgE Unit' was defined as a 1/100 dilution of an internal standard serum pool obtained from HEL (10 μg)/alum (1 mg) sensitized mice.

IL-13, TARC, IFN-γ, MCP-1, KC and eotaxin were determined on BALF fluid supernatant and IL-13, TARC, IFN-γ were measured on the supernatant of cultured lymph node cells using ELISA kits (R&D Systems, Abingdon, UK).

### Statistical analysis

Data were analysed with the statistical packet SPSS 15.0 (SPSS Inc.; Chicago, IL). Values are expressed as mean +/- Standard Error of the Mean (SEM). Groups were compared using the Kruskall-Wallis test for screening significant differences between the groups. When p < 0.05, Mann-Whitney U-test with Bonferroni's conservative corrections were applied to compare the individual groups.

## Results

### 1) Biological effects of repeated exposures to allergen or smoke

In a first experiment, sensitized C57BL/6 mice were challenged with OVA aerosol for 2 weeks to induce an asthma-like Th-2 driven inflammatory response in the airways (Figure 1 – *Allergen Protocol 1A*). Next, we prolonged the exposure to OVA aerosols up to 8 weeks (Figure 1- *Allergen Protocol 1B*). As previously described [3], this treatment made the allergen-induced eosinophilic airway inflammation disappear completely (data not shown).

**Figure 2:**
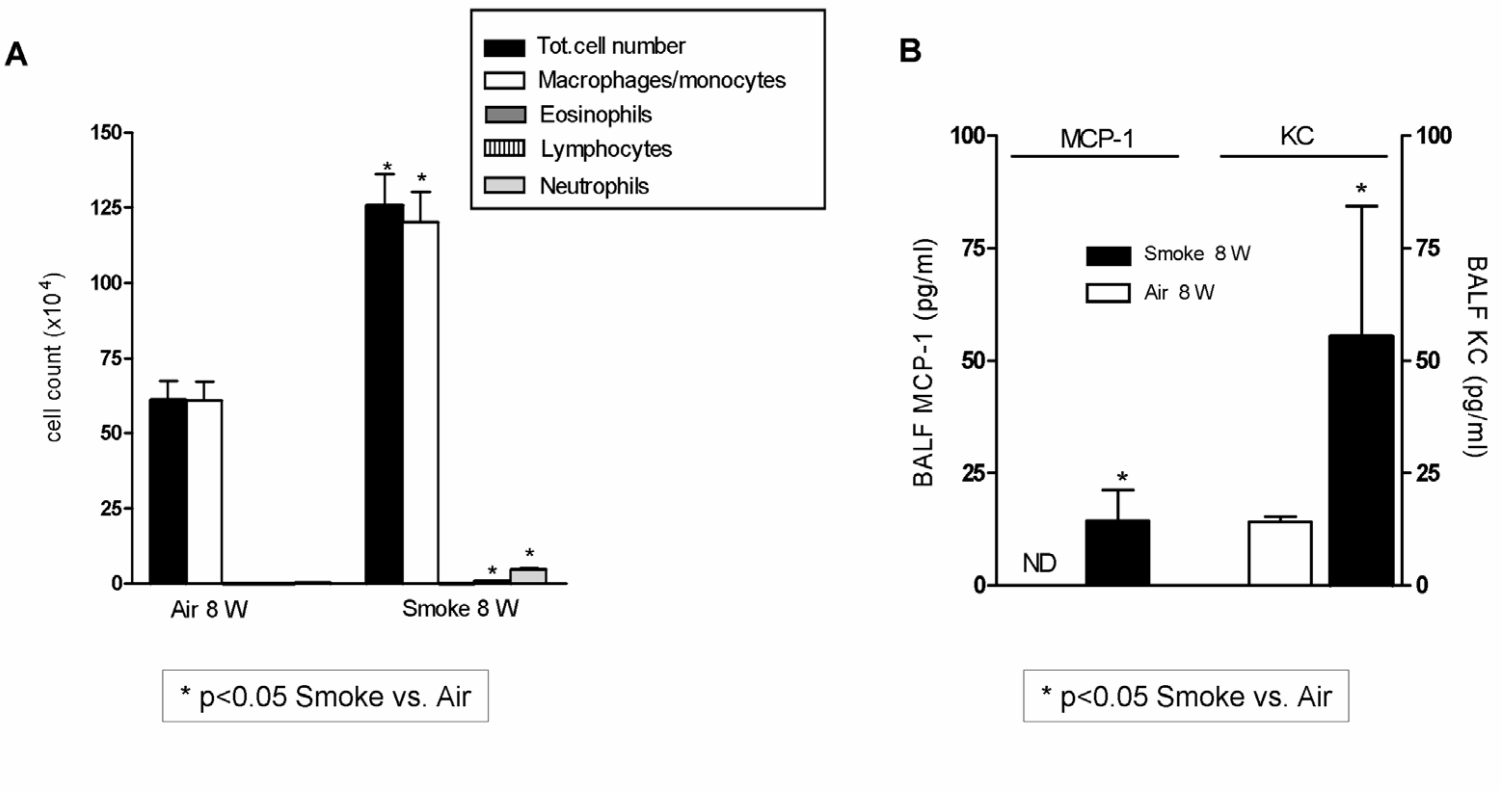
**BALF cell differentiation (A) and BALF cytokines (pg/ml) MCP-1 and KC (B) after 8 week exposure to Smoke or Air in C57BL/6 mice *(Smoke Protocol)***. n = 6 mice/group. * p ≤ 0.05: Smoke vs Air (Mann-Whitney U test); Bars indicate mean ± SEM. ND = Not Detectable (detection limit = 1–2 pg/ml).

Next, to document the biological effect of the applied smoke protocol for 8 weeks, C57BL/6 mice were exposed to either Smoke or Air for 8 weeks (Figure 1- *Smoke Protocol*). Analysis showed that BALF leukocytes, macrophages, neutrophils and lymphocytes were significantly increased in smoke exposed mice (Figure 2A). Furthermore, MCP-1 (Monocyte Chemotactic Protein-1 or CCL2) and KC (cytokine-induced neutrophil chemo-attractant or mouse IL-8, CXCL chemokine) were elevated in BALF supernatant (Figure 2B). Histopathology showed that the bronchi were infiltrated with mononuclear cells and neutrophils after 8 weeks exposure to cigarette smoke (not shown).

### 2) The fading of allergic inflammation by prolonged OVA aerosol challenge is not reversed by cigarette smoke

Next, we evaluated the effects of smoke exposure on the establishment of immune tolerance. Four groups of OVA-sensitized C57BL/6 mice (n = 8/group) were exposed to Smoke or Air combined with OVA or PBS aerosols for 8 weeks (Figure 1- *Combination Protocol 1*).

#### Airway inflammation

**Figure 3:**
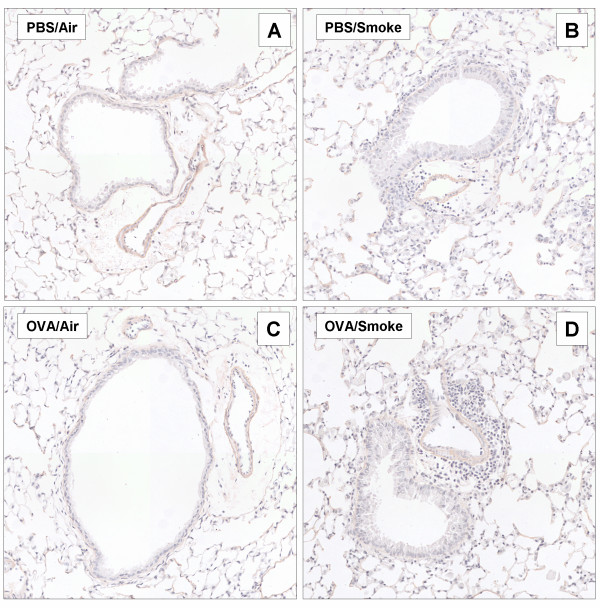
Histology (Congo Red) of the airways of the four groups of mice after 8 week exposure to OVA or PBS aerosols combined with Smoke or Air in sensitized C57BL/6 mice *(Combination Protocol 1) *(Magnification = 400×; A = PBS/Air, B = PBS/Smoke, C = OVA/Air, D = OVA/Smoke).

**Figure 4:**
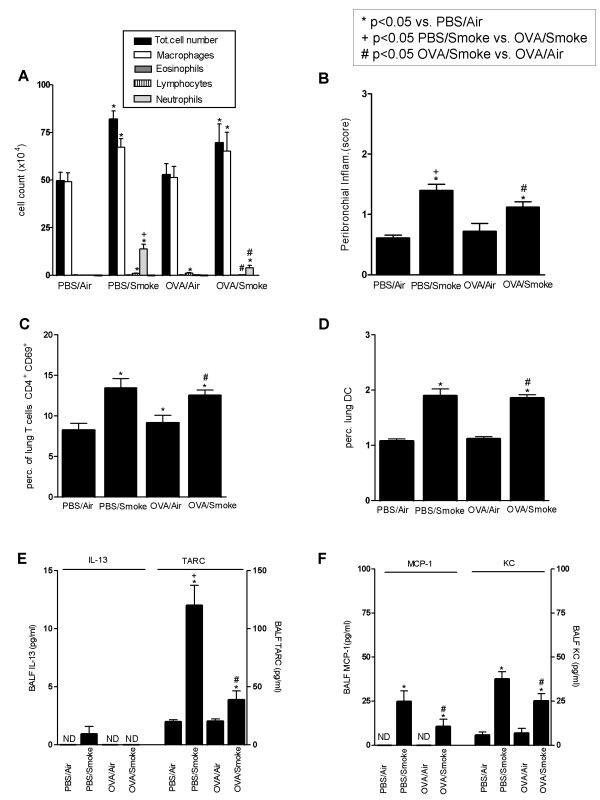
**Results obtained from combining 8 weeks OVA or PBS aerosol exposure with Smoke or Air in sensitized C57BL/6 mice *(Combination Protocol 1)***. (A.) BALF analysis of total cell counts and differentiation after prolonged exposure (8 weeks) to a combination of OVA or PBS aerosols and Smoke or Air. (B) Evaluation of the peribronchial inflammation via a semi-quantitative method (Mean score/airway). (C) Percentage of T – lymphocytes in lung that are CD4^+^CD69^+ ^(as a percentage within the T cell population). (D) Percentage of dendritic cells in digested lung tissue (percentage of total lung cells). (E) Th-2 cytokine (IL-13 and TARC) profiles in BALF (pg/ml). (F) Cytokines MCP-1 and KC in BALF (pg/ml). n = 8 mice/group; bars indicate mean ± SEM. * p ≤ 0.05: all groups vs PBS/Air (Mann-Whitney U test); ^+ ^p ≤ 0.05: PBS/Smoke vs OVA/Smoke (Mann-Whitney U test); ^# ^p ≤ 0.05: OVA/Smoke vs OVA/Air (Mann-Whitney U test); ND = Not Detectable (detection limit = 1–2 pg/ml)

Mice exposed for 8 weeks to OVA inhalations only ('OVA/Air') displayed no detectable airway inflammation. Histology (Figure 3), cytology of BALF and lung (Figure 4A–D), and Th-1/Th-2 cytokines (Figure 4E–F) were comparable between PBS/Air and OVA/Air exposed mice. In contrast, mice exposed to smoke alone without OVA ('PBS/Smoke'), displayed a mild inflammatory response in the airways due to the effects of cigarette smoke, characterized by neutrophils, macrophages and dendritic cells in BALF and lung tissue (Figure 3 and 4). There was an increase in number and percentage of activated T-cells (CD4^+^CD69^+ ^and CD8^+^CD69^+ ^T-cells) in the lung tissue.

In mice exposed to both OVA and smoke ('OVA/Smoke'), we did *not *observe a re-activation of Th-2 driven airway inflammation. In particular, there was no eosinophilic influx in BALF or in the airways as compared to the other groups (Figure 3 and 4A–B). The numbers and percentages of dendritic cells in BALF (data not shown) and lung and activated lung T-cells were comparable with mice exposed to cigarette smoke only (Figure 4C and 4D). Remarkably, the increase in BALF neutrophilia was significantly less pronounced as compared to the mice that were exposed to cigarette smoke only (PBS/Smoke vs OVA/Smoke, p < 0.05), which indicates an attenuated inflammatory response to the smoke in tolerized mice (Figure 4A). Airway histology showed infiltration of mononuclear cells and neutrophils in both smoke -exposed groups, while the infiltration was absent in mice exposed to PBS/Air or OVA/Air. Again this tissue inflammatory reaction was significantly less pronounced in the OVA/Smoke group as compared to the PBS/Smoke group (Figure 3 and 4B).

#### Cytokines

IL-13 in the BALF was around (PBS/Smoke group) or below (other groups) the detection limit (1–2 pg/ml) (Figure 4E). TARC was elevated in response to cigarette smoke, and this increase was less pronounced when OVA and smoke were combined (Figure 4E). MCP-1 and KC were elevated in both cigarette smoke exposed groups, but this increase tended to be lower in the group with combined exposures (For MCP-1: p = 0.083 PBS/Smoke vs OVA/Smoke; for KC: p = 0.065 PBS/Smoke vs OVA/Smoke; Figure 4F). Eotaxin in BALF was below the detection limit in all groups.

In the supernatant of cultured lymph node cells, higher constitutive levels of the Th-2 cytokine IL-13 were measured, but no differences between the groups were found (data not shown). IFN-γ was elevated in supernatant of cultured lymph node cells in response to smoke (data not shown). Again, this increase in IFN-γ was less pronounced in the group were cigarette smoke was combined with OVA aerosols (p < 0.05 PBS/Smoke vs OVA/Smoke).

#### Serum IgE

Total- and OVA-specific serum-IgE were comparably elevated in both OVA-exposed groups.(OVA-IgE: 25.75 ± 8.51 U/ml in OVA/Air vs 35.40 ± 9.47 U/ml in OVA/Smoke; p = 0.38).

### 3) Mainstream cigarette smoke exposure does not reverse the induction of functional bystander tolerance

Next, as combined OVA and mainstream cigarette smoke exposure for 8 weeks induced neither a persistent allergic airway inflammation nor an allergy exacerbation, we evaluated at the functional level *in vivo *whether the tolerance mechanisms effectively remained active. Therefore, we implemented *Allergen protocol 2*, but in the presence of cigarette smoke (Figure 1- *Combination Protocol 2)*. The first group of mice was exposed to 8 weeks of OVA, then re-immunized and re-challenged with HEL ('OVA/HEL/Air' group); the second group was identically treated, but was concurrently exposed to cigarette smoke ('OVA/HEL/Smoke' group).

**Figure 5:**
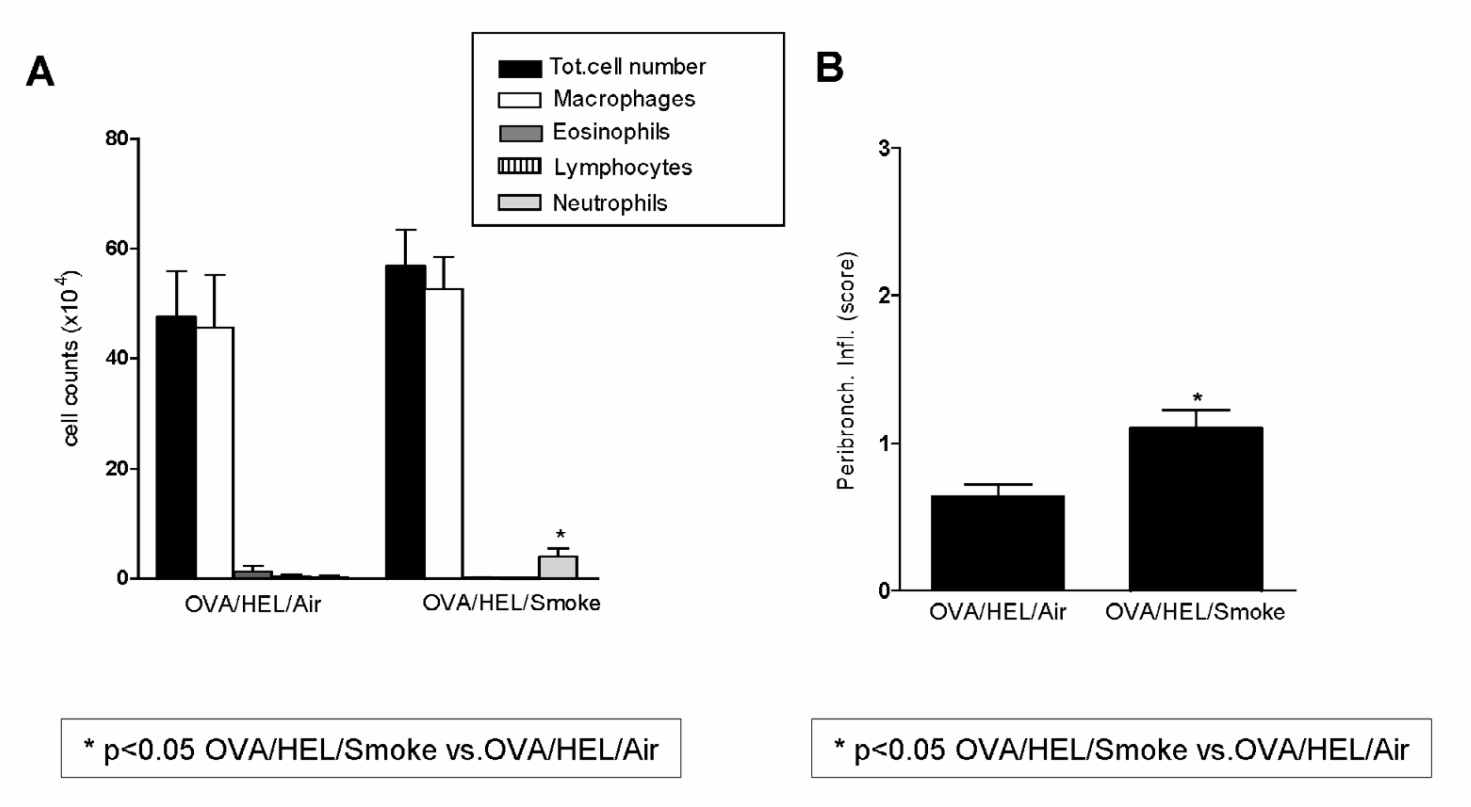
Results obtained 8 weeks of OVA aerosols followed by re-sensitisation (i. p.) and re-challenge to HEL aerosols (*Allergen Protocol 2) *combined with concurrent Smoke or Air-exposures *(Combination Protocol 2)*. (A) BALF cell differentiation. (B) Peribronchial inflammation (Mean score/airway). n = 8 mice/group; bars indicate mean ± SEM. * p ≤ 0.05: OVA/HEL/Smoke vs OVA/HEL/Air (Mann-Whitney U test);

The inflammatory cells in BALF as well as the tissue inflammation and serum HEL-specific IgE (not shown) showed that there was no renewal of Th-2 driven eosinophilic airway disease in the OVA/HEL/Smoke group (Figure 5). This was also underscored by the measurements of Th-1/Th-2 cytokines in BALF (IL-13, TARC and eotaxin were below detection limit in both groups) and in supernatant of cultured lymph node cells (not shown). The only parameters that were different between both groups were neutrophils in BALF and neutrophils and mononuclear cell infiltrates around the airways (histology) in the OVA/HEL/Smoke group, to be attributed to the effects of the tobacco smoke.

### 4) Cigarette smoke exposure enhances the magnitude of an acute inflammatory response and slows down the time course of tolerance induction

Finally, we tested whether combined OVA aerosol and mainstream cigarette smoke exposure could have an influence on the time course of the tolerance induction. In order to investigate this, we concurrently exposed four groups of mice to Smoke or Air and OVA or PBS aerosols for 2 and 4 weeks (Figure 1- *Combination Protocols 3A *and *3B *respectively).

**Figure 6:**
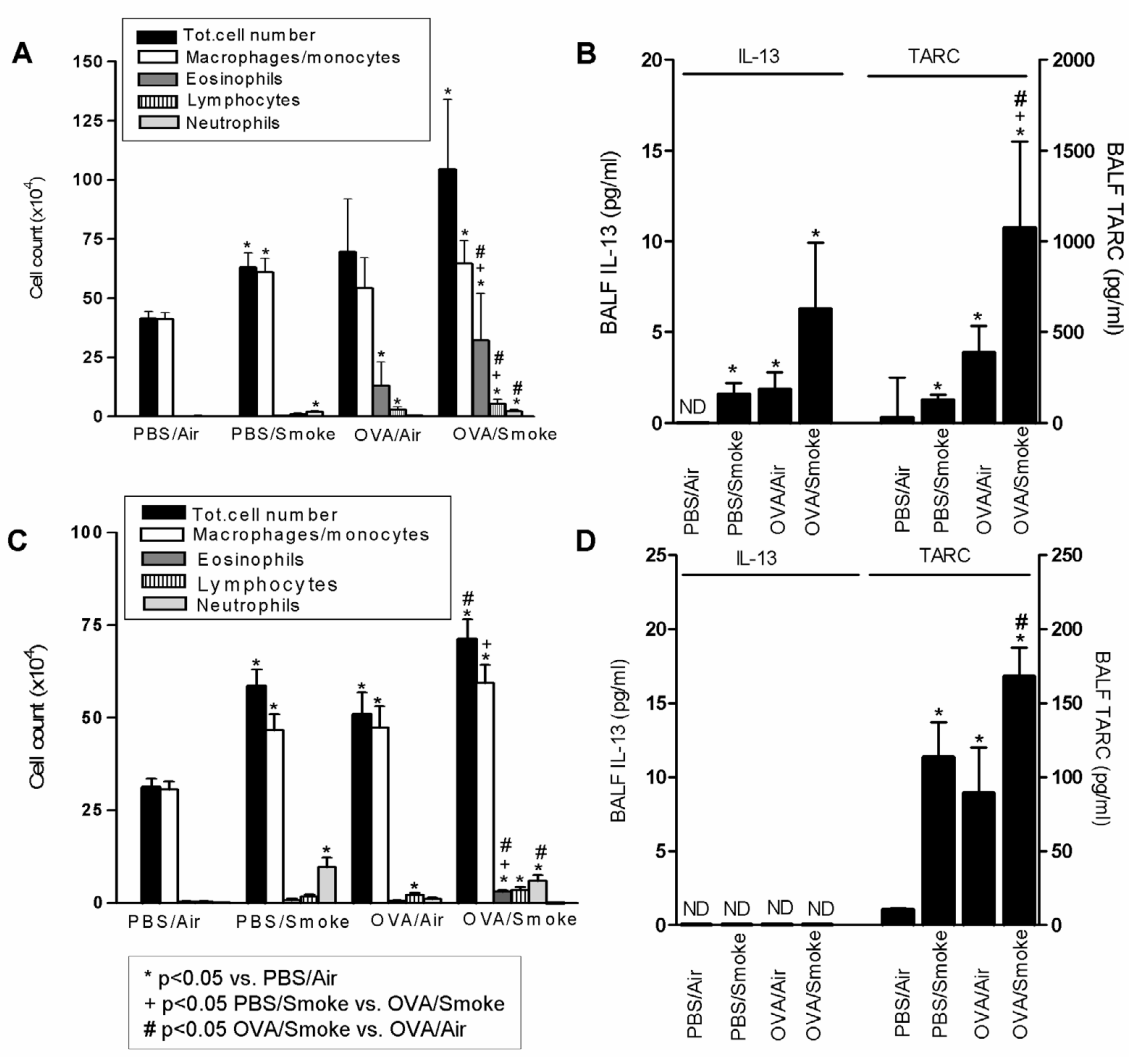
BALF cell differentiation and cytokines (pg/ml) IL-13 and TARC after respectively 2 (Fig. 6A and 6B) and 4 (Fig. 6C and 6D) weeks of concurrent exposure to OVA/PBS and Smoke or Air in C57BL/6 mice *(Combination Protocol 3A and 3B)*. n = 8 mice/group; bars indicate mean ± SEM. * p ≤ 0.05: all groups vs PBS/Air (Mann-Whitney U test); ^+ ^p ≤ 0.05: OVA/Smoke vs. PBS/Smoke (Mann-Whitney U test); ^# ^p ≤ 0.05: OVA/Smoke vs. OVA/Air (Mann-Whitney U test); ND = Not Detectable (detection limit = 1–2 pg/ml)

Combined exposure for 2 weeks (*Combination Protocol 3A*) resulted in an augmented acute Th-2 mediated airway inflammation in OVA/Smoke exposed mice compared with OVA/Air exposed mice. This was evident from BALF analysis (Figure 6A), Th-2 cytokines (Figure 6B), histology (not shown) and lung dendritic cells and activated CD4^+ ^T cells (Table 1). KC and MCP-1 were elevated in BALF of both smoke-exposed groups of mice, while eotaxin was only detectable in both OVA-exposed groups (not shown). CD86 expression on dendritic cells was increased in response to OVA exposure alone, as well as in response to combined OVA/Smoke exposure (Table 1). OVA-IgE tended to be higher in OVA/Smoke compared to OVA/Air exposed mice (Table 1; p = 0.074 OVA/Air vs OVA/Smoke).

**Table 1:** Serum- IgE and Flow Cytometric data

**2 weeks**	**PBS/Air**	**PBS/Smoke**	**OVA/Air**	**OVA/Smoke**
**OVA – IgE (U/ml)**	0.97 ± 0.40	2.30 ± 0.50	14.02 ± 4.44*	20.00 ± 4.38*
**Total IgE (μg/ml)**	0.05 ± 0.01	0.09 ± 0.02	0.52 ± 0.37*	0.56 ± 0.25*
**Lung dendritic cells (perc.)**	0.94 ± 0.06	1.41 ± 0.07*	1.74 ± 0.16*	1.90 ± 0.23*
**Lung CD4**^+^**CD69**^+ ^**T cells (perc. of T cells)**	7.00 ± 1.09	9.69 ± 0.74*	13.88 ± 1.29*	18.52 ± 2.04*^#^
**Lung CD86**^+ ^**dendritic cells (perc. of DC)**	53.27 ± 2.58	59.03 ± 0.96	63.84 ± 2.84*	65.62 ± 2.18*^+^

**4 weeks**	**PBS/Air**	**PBS/Smoke**	**OVA/Air**	**OVA/Smoke**

**OVA- IgE (U/ml)**	2.75 ± 0.79	3.67 ± 0.70	35.23 ± 9.06*	55.22 ± 8.22*
**Total IgE (μg/ml)**	0.17 ± 0.05	0.16 ± 0.03	0.24 ± 0.068	0.33 ± 0.074
**Lung dendritic cells (perc.)**	1.07 ± 1.29	2.09 ± 0.13*^+^	1.33 ± 0.52	1.53 ± 0.86*
**Lung CD4**^+ ^**CD69**^+^**T cells (perc. of T cells)**	8.36 ± 0.90	14.29 ± 1.81*	13.57 ± 0.63*	18.16 ± 1.06*^#^
**Lung CD86**^+^**DC (perc. of DC)**	55.33 ± 3.22	73.21 ± 1.31*	63.07 ± 1.99	72.32 ± 1.17*^#^

n = 8 mice/group; bars indicate mean ± SEM

* p ≤ 0.05: all groups vs PBS/Air (Mann-Whitney U test);

^+ ^p ≤ 0.05: OVA/Smoke vs. PBS/Smoke (Mann-Whitney U test);

^# ^p ≤ 0.05: OVA/Smoke vs. OVA/Air (Mann-Whitney U test);

Next, we investigated the influence of combined exposures on the time course of the tolerance induction by giving the mice both stimuli for 4 weeks (*Combination Protocol 3B)*. The eosinophilic airway inflammation had almost completely disappeared in OVA/Air exposed mice. In contrast, a limited though significant airway eosinophilia remained present in OVA/Smoke mice (Figure 6C). IL-13 was below the detection limit in all groups. TARC (Figure 6D) was elevated in the OVA/Smoke exposed group compared to the OVA/Air group. The eosinophil chemo-attractant eotaxin in BALF was only detectable in the BALF of OVA/Smoke mice (5.20 ± 0.49 pg/ml), while being below the detection limit (<1–2 pg/ml) in the other groups. KC and MCP-1 were elevated in both smoke-exposed groups (data not shown). The observations in BALF were further underscored by measurements of IL-13 and TARC on supernatant of cultured lung-draining lymph node cells and by histology of the airways (not shown). The number of dendritic cells in the lung was elevated in both smoke- exposed groups, while activated CD4^+ ^T cells were elevated in the OVA/Smoke group as compared to mice exposed to OVA alone (Table 1). The percentage of CD86 positive lung dendritic cells was significantly increased compared to PBS/Air mice in response to smoke alone and OVA/Smoke, but not to OVA alone (Table 1). Serum OVA-IgE remained elevated in both OVA challenged groups, but cigarette smoke exposure had, as for 2 weeks, no significant additive effect (Table 1; p = 0.40, OVA/Air vs OVA/Smoke).

**Figure 7:**
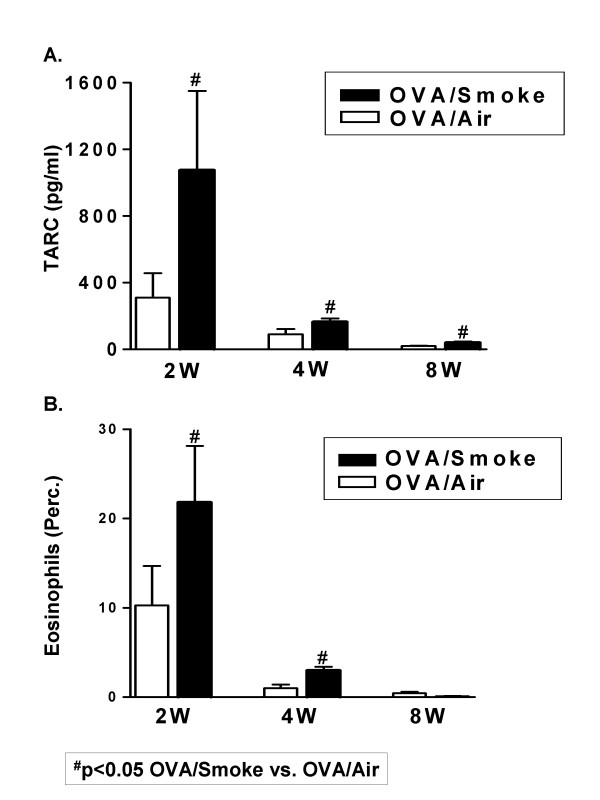
The evolution in the levels of TARC in BALF (pg/ml) and the percentage of eosinophils in BALF over time after 2, 4 and 8 weeks of concurrent exposure to OVA/PBS and to Smoke or Air in C57BL/6 mice *(Combination Protocol 1, 3A and 3B)*. ^# ^p ≤ 0.05: OVA/Smoke vs. OVA/Air (Mann-Whitney U test);

Figure 7 represents a time curve to summarize these findings. We selected two important parameters, namely BALF eosinophilia and TARC. This figure illustrates the effects of combined cigarette smoke and OVA aerosol exposure for 2, 4 and 8 weeks. Figure 7 shows that cigarette smoke exposure initially augmented airway eosinophilia and BALF TARC levels and later on slowed down the onset of tolerance.

## Discussion

The objective of this study was to evaluate the effects of mainstream cigarette smoke in a mouse model of inhalational tolerance. We show that a state of disease inhibition or 'tolerance', as we refer to this state of unresponsiveness to re-sensitization and re-challenge, can be induced in mice suffering allergic airway inflammation, despite concurrent exposure to both mainstream cigarette smoke and inhaled allergen. The smoke – induced inflammation even appeared attenuated in these tolerized mice. Importantly, mainstream cigarette smoke enhanced the acute allergic inflammation and slowed down the time course of tolerance induction.

We combined the 8 weeks OVA aerosol exposure protocol, sufficient to induce tolerance in sensitized C57BL/6 mice, with cigarette smoke *(Combination Protocol 1) *and found that cigarette smoke did *not *prevent the establishment of tolerance due to prolonged OVA aerosol challenge. In general, functional tolerance *in vivo *can be proven by re-immunization and challenge with the same or with bystander allergens. This experimental approach has been used to prove primary inhalational tolerance [22] or oral tolerance [23] in naïve mice. We previously also applied the same approach to this model of prolonged OVA aerosol challenge and we discovered that, after the eosinophilic inflammation had disappeared, re-immunization and short-term allergen re-challenge did not result in the re-appearance of allergic airway disease, and that this phenomenon had memory characteristics [3]. Here, re-immunization and re-challenge of the mice with the secondary antigen HEL in the presence of cigarette smoke *(Combination Protocol 2) *additionally showed that, also at the functional level, no Th-2 mediated immune responses could be mounted, despite the cigarette smoke. In line with these findings, one recent study also found no evidence of increased allergic inflammatory responses in a model of combined long-term OVA and tobacco smoke exposure, although another method of tobacco smoke exposure (nose-only) was used [24].

Remarkably, some parameters indicated that the inflammatory response to smoke was even attenuated in the mice concurrently subjected to the 8 weeks inhalational tolerance protocol. These findings suggest that the tolerance mechanisms could also dampen the airways' response to other pro-inflammatory stimuli or airway irritants. Since one of the proposed mechanisms is the induction of regulatory T cell activity, it is interesting to note that allergen-induced regulatory T cells can inhibit both Th-2 [25] and Th-1 responses *in vivo*, while they can also dampen the activation of the innate immune system [1]. However, in contrast with primary inhalational tolerance in naïve animals, where an important role for regulatory T cells has been documented [22,26], we previously could not find any increases in the numbers of 'naturally occurring' CD4^+^CD25^+^Foxp3^+ ^regulatory T cells in lung tissue upon chronic antigen inhalation [3]. Although this finding did not preclude the induction of other regulatory T cells subsets and gave no definite answer about the activity of these cells, it is tempting to speculate that regulatory T cell-independent mechanisms might be involved, such as induction of anergy, depletion of the T cells, and immunosuppressive activity of alveolar macrophages. In support of this hypothesis, inhibition of immune responses by repeated allergen challenge could also be obtained in one model in the absence of regulatory T cells, as shown by adoptive transfer experiments in RAG1^-/- ^mice [27]. Recent data also showed the involvement of complement factor C5a in inhalational tolerance in naïve mice [28], although conflicting results have been published about its precise role during the course of an allergic inflammatory response [28-30]. Consequently, the role of the complement in inducing and/or maintaining inhalational tolerance through prolonged allergen challenge in sensitized mice has to be delineated further.

Although we had to reject our primary hypothesis that cigarette smoke could prevent tolerance establishment, we here documented that cigarette smoke exposure could influence the kinetics of the tolerance induction. Firstly, 2 weeks of concurrent exposure *(Combination Protocol 3A) *augmented the acute allergic airway inflammation in experimental asthma. This confirmed our previous observations of the aggravating effects of cigarette smoke on acute allergic inflammation in a BALB/c model of acute allergic airway inflammation [17]. In this model, a significant increase in OVA-IgE was also observed in response to concurrent short-term OVA and smoke exposure, while here only a trend was observed. These differences in response can be attributed to the fact that the BALB/c strain is naturally a high-IgE responder [31], in contrast to the C57BL/6 strain used here, which is lower-IgE responder to systemic sensitization. Secondly, combined exposures for 4 weeks *(Combination Protocol 3B) *showed that cigarette smoke significantly slowed down the onset of tolerance. We found persistence of airway eosinophilia and Th-2 mediators in OVA/Smoke mice but not in OVA/Air mice. This result clearly showed that tobacco smoke could influence the time course of the tolerance establishment.

A potential underlying mechanism involved in these phenomena is that mainstream tobacco smoke could exert pro-allergic effects by enhancing the maturation status and migration of the dendritic cells in the airways, either directly via its effects on the sub-epithelial dendritic cells [32,33] or indirectly via stimulating the airway epithelium to release dendritic cell maturation stimuli such as TSLP and GM-CSF [34]. Over-expression of these growth factors in the airways have been shown to break inhalational tolerance [4,35,36]. Recently, a more mature dendritic cell phenotype has also been found in smokers [37]. On the other hand, inhibition of dendritic cell maturation in response to smoke has also been described in other studies using different exposure protocols [38], or *in vitro *conditioning of human dendritic cells with cigarette smoke extract [39], as well as in some human studies [40]. Although we here found a significant increase in the expression of CD86 on pulmonary dendritic cell after 4 weeks of OVA/Smoke exposure, the exact role of the dendritic cell maturation status and migration to the lymph nodes in the exacerbated acute inflammation and the delay in tolerance induction remains to be established.

It is also possible that other cigarette smoke -related factors can co-determine the immune responses in our model at the various time points, either directly or indirectly by influencing the dendritic cells. Lipopolysaccharide (LPS) is, among other danger signals, abundantly present in tobacco smoke. The effects of LPS on the immune system in general, and on the dendritic cell-T cell axis in particular, are dose dependent. As such, it was shown that low doses of LPS favour Th-2 responses whereas higher dosages usually suppress Th-2 responses in both humans and mice [41-45]. Concurrent allergen and cigarette smoke for longer time periods could thus have totally different effects on the eosinophilic inflammation than concurrent short-term exposures.

Relating to the clinic, it still remains to be established whether prolonged immune stimulation by the continuous 'therapeutic' administration of allergen in order to either exhaust allergen specific Th-2 memory cell populations or induce (regulatory T cell mediated) tolerance is a feasible approach [46]. Nevertheless, our data here indicate that cigarette smoke could interfere with such approaches.

## Conclusion

This study shows that allergen-induced tolerance can be established in experimental asthma despite concurrent mainstream tobacco smoke exposure. Nevertheless, tobacco smoke exposure enhances the acute allergic airway inflammation and slows down the induction of tolerance, resulting in a more persistent eosinophil-rich airway inflammation.

## List of abbreviations

OVA: Ovalbumin; HEL: Hen Egg Lysozyme; PBS: Phosphate-Buffered Saline; BALF: Broncho-alveolar Lavage Fluid; TCM: Tissue Culture Medium; ELISA: Enzyme-Linked Immunosorbent Assay; TARC: Thymus-and Activation Regulated Chemokine; MCP-1: Monocyte Chemotactic Protein-1; KC: cytokine-induced neutrophil chemoattractant; TSLP: Thymic Stromal Lymphopoietin; GM-CSF: Granulocyte-Macrophage Colony Stimulating Factor; LPS: Lipopolysaccharide

## Competing interests

The authors declare that they have no competing interests.

## Authors' contributions

CVH and KT were involved in the conception and design of the studies. CVH and KM carried out the laboratory experiments under supervision of TM. CVH, GJ and KT drafted the manuscript. All authors approved the final manuscript.
